# Neonatal Periostin Knockout Mice Are Protected from Hyperoxia-Induced Alveolar Simplication

**DOI:** 10.1371/journal.pone.0031336

**Published:** 2012-02-17

**Authors:** Paul D. Bozyk, J. Kelley Bentley, Antonia P. Popova, Anuli C. Anyanwu, Marisa D. Linn, Adam M. Goldsmith, Gloria S. Pryhuber, Bethany B. Moore, Marc B. Hershenson

**Affiliations:** 1 Department of Pediatrics and Communicable Diseases, University of Michigan Medical School, Ann Arbor, Michigan, United States of America; 2 Department of Internal Medicine, University of Michigan Medical School, Ann Arbor, Michigan, United States of America; 3 Department of Molecular and Integrative Physiology, University of Michigan Medical School, Ann Arbor, Michigan, United States of America; 4 Department of Microbiology and Immunology, University of Michigan Medical School, Ann Arbor, Michigan, United States of America; 5 Department of Pediatrics, University of Rochester School of Medicine and Dentistry, Rochester, New York, United States of America; Louisiana State University, United States of America

## Abstract

In bronchopulmonary dysplasia (BPD), alveolar septae are thickened with collagen and α-smooth muscle actin, transforming growth factor (TGF)-β-positive myofibroblasts. Periostin, a secreted extracellular matrix protein, is involved in TGF-β-mediated fibrosis and myofibroblast differentiation. We hypothesized that periostin expression is required for hypoalveolarization and interstitial fibrosis in hyperoxia-exposed neonatal mice, an animal model for this disease. We also examined periostin expression in neonatal lung mesenchymal stromal cells and lung tissue of hyperoxia-exposed neonatal mice and human infants with BPD. Two-to-three day-old wild-type and periostin null mice were exposed to air or 75% oxygen for 14 days. Mesenchymal stromal cells were isolated from tracheal aspirates of premature infants. Hyperoxic exposure of neonatal mice increased alveolar wall periostin expression, particularly in areas of interstitial thickening. Periostin co-localized with α-smooth muscle actin, suggesting synthesis by myofibroblasts. A similar pattern was found in lung sections of infants dying of BPD. Unlike wild-type mice, hyperoxia-exposed periostin null mice did not show larger air spaces or α-smooth muscle-positive myofibroblasts. Compared to hyperoxia-exposed wild-type mice, hyperoxia-exposed periostin null mice also showed reduced lung mRNA expression of α-smooth muscle actin, elastin, CXCL1, CXCL2 and CCL4. TGF-β treatment increased mesenchymal stromal cell periostin expression, and periostin treatment increased TGF-β-mediated DNA synthesis and myofibroblast differentiation. We conclude that periostin expression is increased in the lungs of hyperoxia-exposed neonatal mice and infants with BPD, and is required for hyperoxia-induced hypoalveolarization and interstitial fibrosis.

## Introduction

Increased survival of very premature infants has been accompanied by an increased incidence of bronchopulmonary dysplasia (BPD) [1]. In the “new BPD,” there are larger and fewer alveoli, as well as poorly formed secondary crests, indicating interference with septation [2], [3]. Alveolar septa are thickened with collagen and α-smooth muscle actin-, transforming growth factor (TGF)-β-positive myofibroblasts [4], [5], [6], [7]. Adenoviral transfer of the TGF-β gene to newborn rat lungs induces changes consistent with BPD, including excess matrix deposition and large undeveloped pre-alveolar saccules [8]. Overexpression of TGF-β in neonatal mouse lungs induces proliferation of α-actin-positive cells within the alveolar septal walls and hypoalveolarization [9]. Together, these data imply a critical role for TGF-β in the development of BPD.

We have isolated mesenchymal stromal cells from the tracheal aspirates of premature infants [10]. Primary cell colonies produce TGF-β1 and undergo TGF-β-induced myofibroblastic differentiation, suggesting that, in the absence of other signals, myofibroblastic differentiation represents the “default program” for mesenchymal stromal cell specialization [11]. Isolation of these cells is associated with the development of BPD [12]. Gene expression profiling revealed that, compared to lung fibroblasts, mesenchymal stromal cells overexpress the gene *POSTN* [13]. *POSTN* encodes periostin, a secreted protein with an N-terminal secretory signal sequence and four fasciclin domains. Periostin, a member of a subset of non-structural extracellular matrix-associated molecules termed “matricellular proteins,” directly interacts with other extracellular matrix proteins including collagen and fibronectin and is a ligand for α_v_β_3_, α_v_β_5_ and α_4_β_6_ integrins. In the lung, periostin is expressed in stromal cells surrounding squamous cell carcinoma [14], rat pulmonary artery smooth muscle cells [15], primary human lung fibroblasts [16] and human bronchial epithelial cells [17], [18]. Periostin treatment increases TGF-β1 mRNA expression in human bronchial epithelial cells as well as TGF-β1-mediated collagen I gene expression in airway fibroblasts [18]. In the heart, periostin is induced by TGF-β but also required for normal TGF-β responsiveness [19]. Periostin promotes myofibroblast differentiation of palmar fascia mesenchymal cells [20] and is a component of subepithelial fibrosis in asthma [21]. Lysyl oxidase, which crosslinks collagen and elastin, is proteolytically activated by periostin [22], [23]. Together, these data suggest that periostin plays a significant role in TGF-β-mediated fibrosis and myofibroblast differentiation.

Based on the above evidence that TGF-β plays an important role in the pathogenesis of BPD, we hypothesized that periostin expression is required for the myofibroblastic differentiation and alveolar simplication in hyperoxia-exposed neonatal mice, a commonly used animal model for this disease. We also examined the effects of periostin on mesenchymal stromal cell myofibroblastic differentiation *in vitro*, as well as the expression of periostin in BPD lungs.

## Results

### Hyperoxia induces a BPD phenotype in neonatal mice

**Figure 1:**
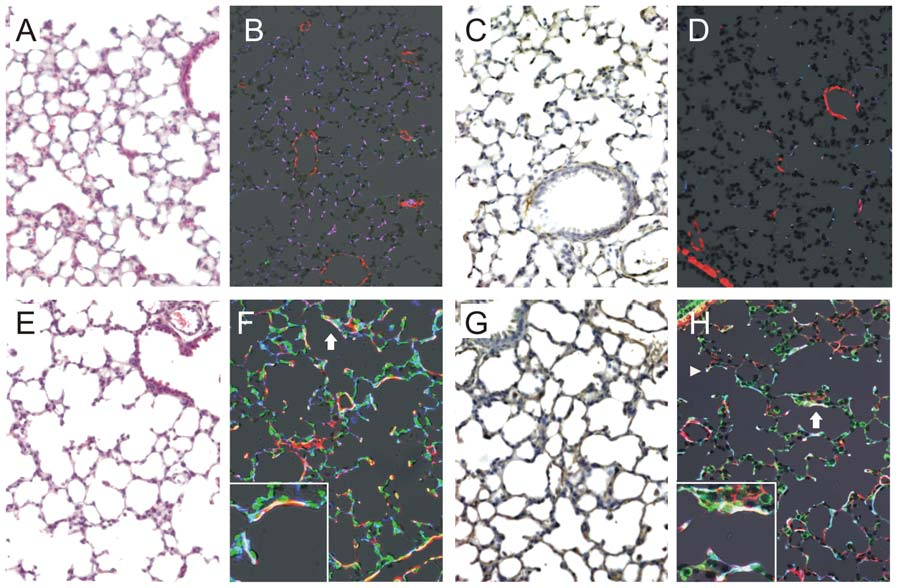
Hyperoxia induces a BPD phenotype in neonatal mice. Two-to-three day-old wild-type C57BL/6J mice were exposed to air or 75% oxygen for 14 days. Compared to air-exposed mice (panels A–D). hyperoxic exposure caused the development of fewer and larger airspaces (E). Fluorescence microscopy showed increased deposition of α-actin (red), elastin (green) and collagen-I (blue, F). Colocalization of α-actin, elastin and collagen-I appears white (arrow, inset). G. Immunohistochemical stains showed periostin expression in the alveolar walls, particularly in areas of interstitial thickening. H. Periostin expression (green) colocalized with α-smooth muscle actin (red) and collagen (blue), suggesting synthesis by myofibroblasts Colocalization of periostin and collagen appears light blue; colocalization of α-actin, periostin and collagen-I appears white (arrow, inset). Colocalization was also present at the tips of secondary crests (arrowhead). Original magnification, 200×. These results are typical of three individual experiments.

Two-to-three day-old wild-type C57BL/6J mice were exposed to air or 75% oxygen for 14 days, as described [18]. This period corresponds to the alveolar stage of mouse lung development, corresponding to the 28–32 week human fetus [24]. As shown previously [25], compared to air-exposed mice (panels 1A–D), hyperoxic exposure caused the development of fewer and larger airspaces (Figure 1E). Analogous to BPD, lungs of hyperoxia-exposed mice showed increased elastin and collagen-I deposition by α-actin-positive myofibroblasts (Figure 1F). Hyperoxia also increased lung periostin expression. In the lungs of air-exposed mice, immunohistochemical staining revealed a modest amount of periostin expression in the airway tissues, as well as the tips of developing alveolar walls (Figure 1C). In the lungs of hyperoxia-exposed mice, there was increased periostin expression in the alveolar walls, particularly in areas of interstitial thickening (Figure 1G). In some instances, periostin co-localized with α-smooth muscle actin, suggesting synthesis by myofibroblasts (Figure 1H). Sections stained with isotype control showed no signal (not shown).

**Figure 2:**
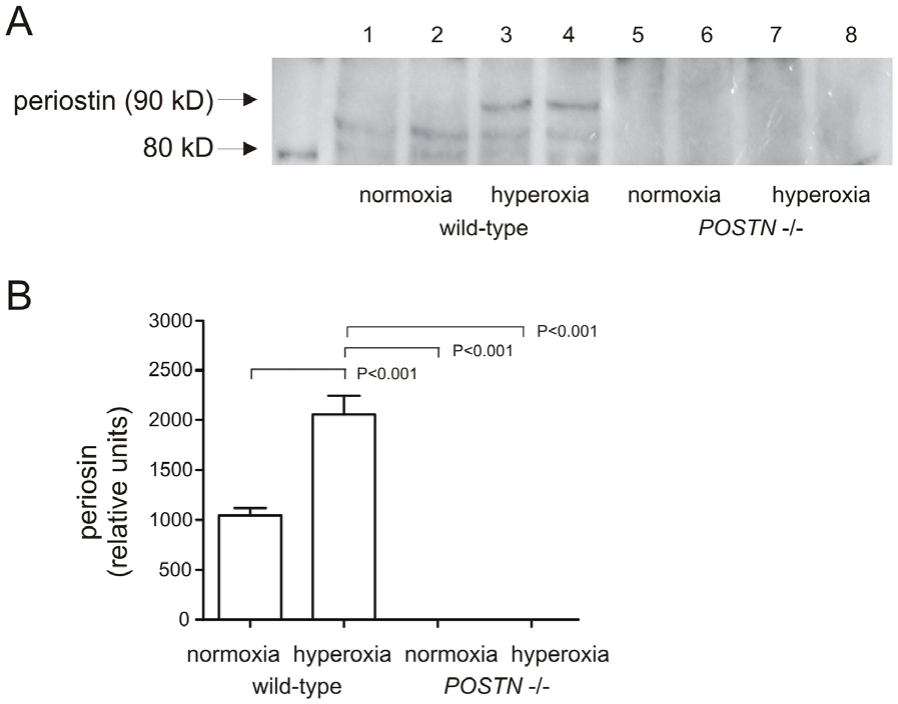
Hyperoxic exposure on neonatal mice increases lung periostin content. Whole lung lysates were resolved by SDS-PAGE, transferred to nitrocellulose and probed with anti-periostin (A). Results from two normoxic wild-type mice and two hyperoxic wild-type mice are shown here; lysates from normoxic and hyperoxic periostin null mice are added for comparison. Immunoblots showed a full-length 90 kD isoform as well as two smaller bands. (B). Group mean data showing a significant increase in periostin expression with hyperoxic exposure (n = 4 for each group, one way ANOVA).

We also analyzed whole lung extracts for periostin protein expression by immunoblotting (Figure 2). The full-length periostin protein is 90 kD in size, but there are also products of splice variants [26]. Hyperoxic exposure increased periostin protein abundance over two-fold (p<0.05, one-way ANOVA).

### Increased periostin expression in the lungs of infants with BPD

**Figure 3:**
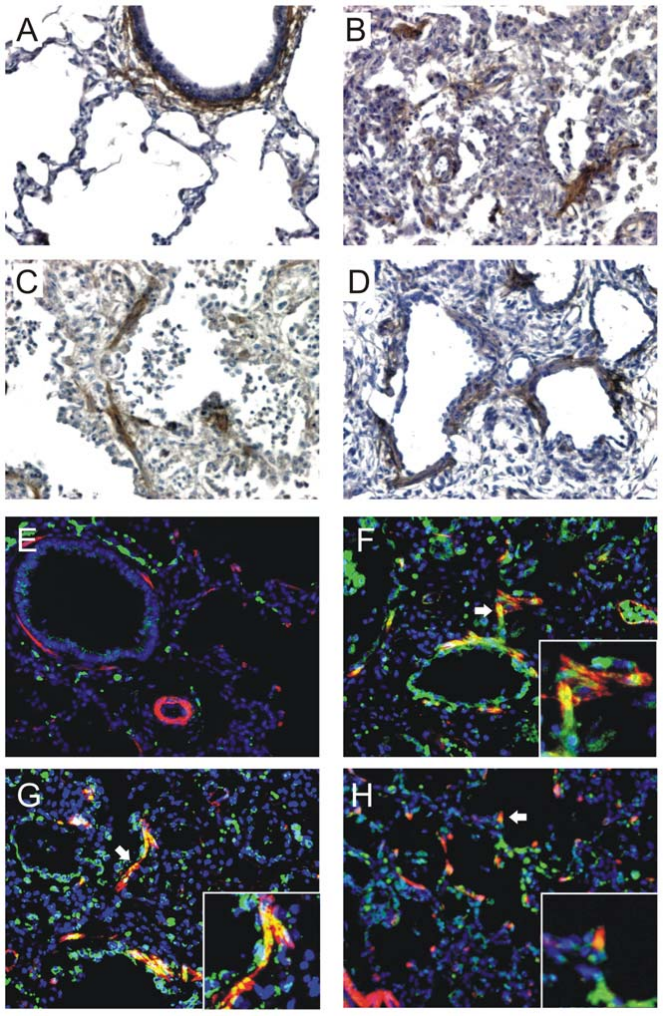
Increased periostin expression in the lungs of infants with BPD. The lung of a full-term infants dying of a non-pulmonary cause is shown in (A). There is significant staining in the airway subepithelium, with miminal staining of the airway epithelium or alveolar walls. B–D. Staining of lung sections from three individual infants dying of BPD showed increased periostin expression, particularly in the subepithelium and fibroblastic foci. E. We also examined periostin (green) and α-smooth muscle actin (red) expression by fluorescence microscopy. Lungs of full-term infants showed periostin expression in the airway subepithelium which was distinct from the adjacent smooth muscle. F–H. Lungs of three individual infants with BPD were also examined for periostin expression. Lungs showed colocalization of periostin and α-actin in interstitial alveolar myofibroblasts (F and G, arrows, insets). Colocalization of periostin and α-actin (yellow-orange) was also found at the tips of secondary crests (H, arrow, inset). Original magnification, 200×.

Periostin staining of lung sections from infants dying of BPD were compared with those from full-term infants. Immunohistochemical staining in the lungs of full-term infants was localized to the airway subepithelium (Figure 3A). Staining in BPD lungs was increased, and was most prominent in the subepithelium of thickened alveolar walls (Figures 3B–D). There appeared to be some staining in fibroblastic foci. Using florescence microscopy, lungs of full-term infants showed periostin expression in the airway subepithelium which was distinct from the adjacent smooth muscle (Figure 3E). In BPD lungs, there is colocalization of α-smooth muscle actin and periostin in interstitial fibroblasts and at the tips of secondary crests (Figures 3F–H).

### Periostin knockout prevents hypoventilation and myofibroblast differentiation in hyperoxia-exposed neonatal mice

**Figure 4:**
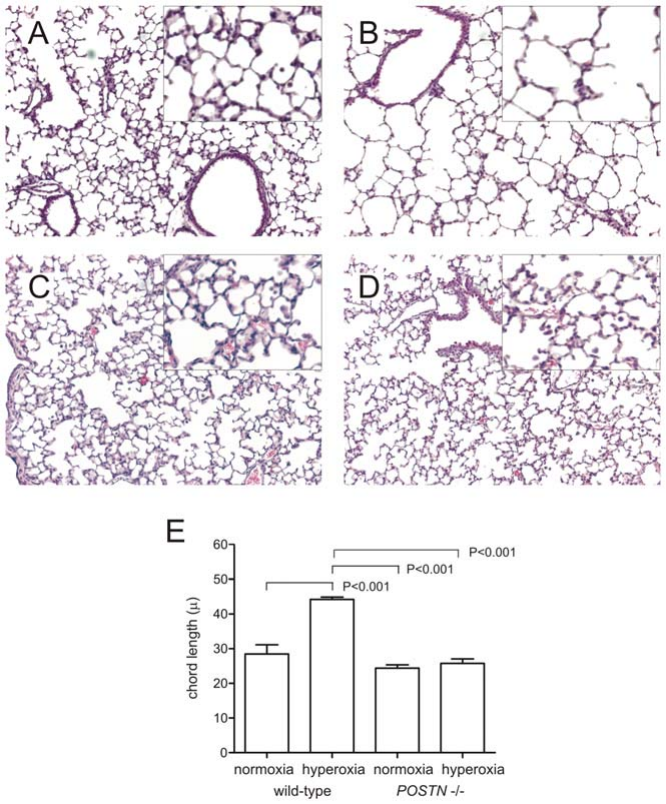
Periostin knockout prevents hypoventilation and myofibroblast differentiation in hyperoxia-exposed neonatal mice. Two-day-old periostin null mice were exposed to air or 75% oxygen for 14 days. Compared to air-exposed wild-type mice (A), hyperoxia-exposed wild-type mice showed alveolar simplication (panel B). In contrast, air- (C) and hyperoxia-exposed periostin null mice (D) showed normal alveolar architecture. (E). Hypoalveolarization in wild-type hyperoxia-exposed mice was associated with a statistically significant increase in mean alveolar chord length (n = 4, one-way ANOVA).

Two-to-three day-old B6;129-*Postn^tm1Jmol^*/J mice were exposed to air or 75% oxygen for 14 days. Air-exposed wild-type mice showed a normal alveolarization pattern (Figure 4A). In contrast, hyperoxic exposure of wild-type mice caused alveolar simplification (Figure 4B). Air-exposed periostin null mice showed a normal alveolarization pattern. Close examination appeared to show a slight increase in the cellularity of alveolar walls, and increased alveolar macrophages (Figure 4C). Unlike wild-type mice, hyperoxia-exposed periostin null mice did not show larger air spaces (Figure 4D). In hyperoxia-exposed wild-type mice, hypoalveolarization was associated with an increase in mean alveolar chord length (Figure 4E).

**Figure 5:**
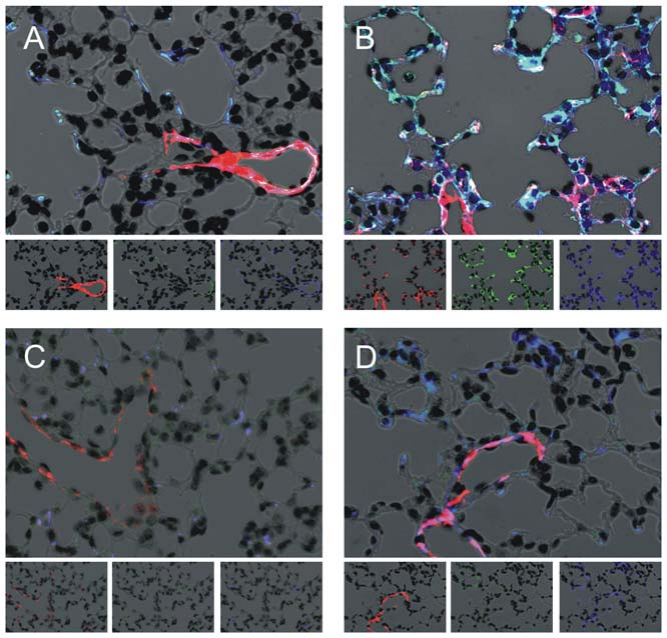
Hyperoxic exposure is associated with α-actin and periostin-double positive myofibroblasts in wild-type but not periostin null mice. Lung sections were stained for α-actin (red), periostin (green) and collagen I (blue); colocalization appears white. Unlike air-exposed wild-type mice (panel A), hyperoxia-exposed wild-type mice showed thickening of the interstitial space with α-smooth muscle-, periostin- and collagen type I-positive myofibroblasts (B). Air- (C) and hyperoxia-exposed periostin null mice (D) did not show alveolar myofibroblasts. These results are typical of three individual experiments.

Air-exposed wild-type mice showed α-smooth muscle actin staining (red) in the airway and arterial walls, but no expression in the alveolar interstitia (Figure 5A). Hyperoxia-exposed wild-type mice showed thickening of the interstitial space with α-smooth muscle-positive myofibroblasts (Figure 5B, α-actin, red; periostin, green; collagen I, blue; colocalization is white). Neither air- nor hyperoxia-exposed periostin null mice showed α-actin-positive myofibroblasts, interstitial thickening or lung periostin expression (Figures 5C and 5D).

### Effects of hyperoxia and periostin knockout on lung mRNA expression

**Figure 6:**
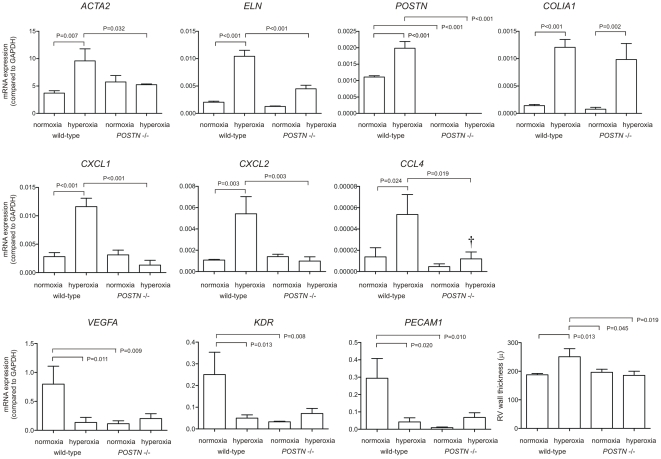
Effects of hyperoxia on lung mRNA expression in wild-type and periostin null mice. mRNA was measured by quantitative PCR. We examined mRNA expression of *ACTA2*, *ELN*, *POSTN*, *COLIA1*, *CXCL1*, *CXCL2*, *CCL4*, *VEGFA*, *KDR* and *PECAM1* (n = 4, one-way ANOVA). Mice were also analyzed by right ventricular wall thickness (n = 4–6, one way ANOVA).

We examined the effects of periostin knockout on lung mRNA expression, as measured by quantitative PCR. Consistent with the observed increase in periostin-positive interstitial myofibroblasts, hyperoxic exposure significantly increased the expression of α-smooth muscle actin, elastin and periostin (Figure 6, each p<0.05, one-way ANOVA). There was no significant increase in α-actin, elastin or periostin expression in the hyperoxia-exposed periostin null mice. Periostin KO mice did not show a reduction in hyperoxia-induced collagen type Iα1 expression. Hyperoxia also significantly increased the mRNA expression of three chemokines – CXCL1, CXCL2 and CCL4 – in wild-type but not periostin null mice. Three genes related to angiogenesis – those encoding vascular endothelial growth factor (VEGF)-A, KDR/VEGF-receptor 2/Flk1 and platelet endothelial cell adhesion molecule (PECAM)-1/CD31 – were significantly downregulated by hyperoxia treatment. However, the expression of these genes was significantly reduced in air-exposed periostin knockout mice, and hyperoxia had no effect on the expression of these genes.

We also examined right ventricular thickness in air- and hyperoxia-exposed wild-type and periostin null mice (Figure 6). As reported previously [27], hyperoxia increased right ventricular wall thickness, suggestive of pulmonary hypertension (p<0.05, one way ANOVA). There was no increase in wall thickness in periostin null mice.

### TGF-β treatment of human mesenchymal stromal cells induces periostin mRNA and protein expression

**Figure 7:**
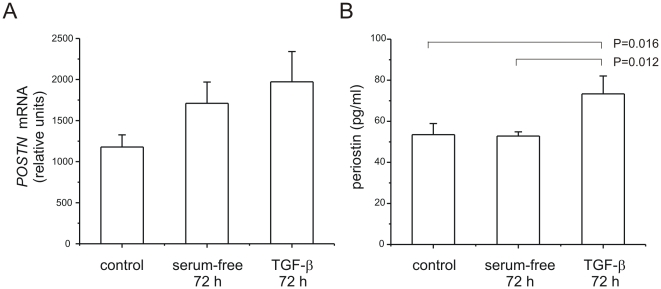
TGF-β treatment of mesenchymal stromal cells induces periostin expression. Neonatal lung mesenchymal stromal cells were treated with 10 ng/ml TGF-β for 72 h and periostin mRNA and protein expression assessed by qPCR and ELISA, respectively. A. mRNA expression tended to increase with TGF-β treatment (n = 4). B. TGF-β treatment significantly increased periostin protein abundance (n = 7, one-way ANOVA).

TGF-β treatment has been shown to increase periostin expression in cardiac myocytes, aortic smooth muscle, and bronchial epithelium. We treated neonatal lung mesenchymal cells with 10 ng/ml TGF-β for 72 h and assessed periostin mRNA and protein expression by qRT-PCR and ELISA, respectively. TGF-β treatment tended to increase periostin mRNA levels, but the changes were not statistically significant (Figure 7A). Mesenchymal stromal cells expressed pg levels of periostin protein at baseline, and there was a small but statistically significant increase with TGF-β treatment (Figure 7B).

### Periostin induces human mesenchymal stromal cell DNA synthesis

**Figure 8:**
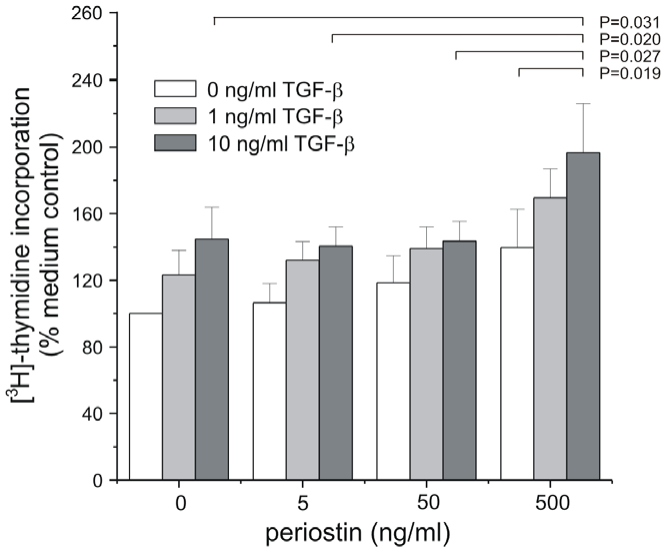
Effects of periostin and TGF-β on mesenchymal stromal cell DNA synthesis. Mesenchymal stromal cells (n = 4) were incubated with [^3^H]-thymidine and treated with either TGF-β or periostin. [^3^H]-thymidine incorporation was assessed by scintillation counting. We used two-way ANOVA with Fisher's least significant difference multiple comparison test to assess the individual and combined effects of TGF-β and periostin on neonatal lung mesenchymal stromal cell DNA synthesis. In the presence of 500 ng/ml periostin, 10 ng/ml TGF-β significantly increased DNA synthesis compared to periostin alone. Also, in the presence of 10 ng/ml TGF-β, 500 ng/ml periostin significantly increased DNA synthesis compared to other periostin concentrations.

We examined the interactive effects of periostin (0–500 ng/ml) and TGF-β (0–10 ng/ml) on cellular [^3^H]-thymidine incorporation by two-way ANOVA (Figure 8). We employed concentrations of periostin found to present in fibrotic lesions *in vivo* [20]. Cells treated with 10 ng/ml TGF-ß in the presence of 500 ng/ml periostin showed an increase in DNA synthesis, whereas cells treated with 10 ng/ml TGF-β in the absence of periostin did not show an increase. Similarly, cells treated with 500 ng/ml periostin in the presence of 10 ng/ml TGF-β showed an increase in DNA synthesis, whereas cells treated with 500 ng/ml periostin in the absence of TGF-β did not.

### Periostin enhances TGF-β-induced myofibroblastic differentiation

**Figure 9:**
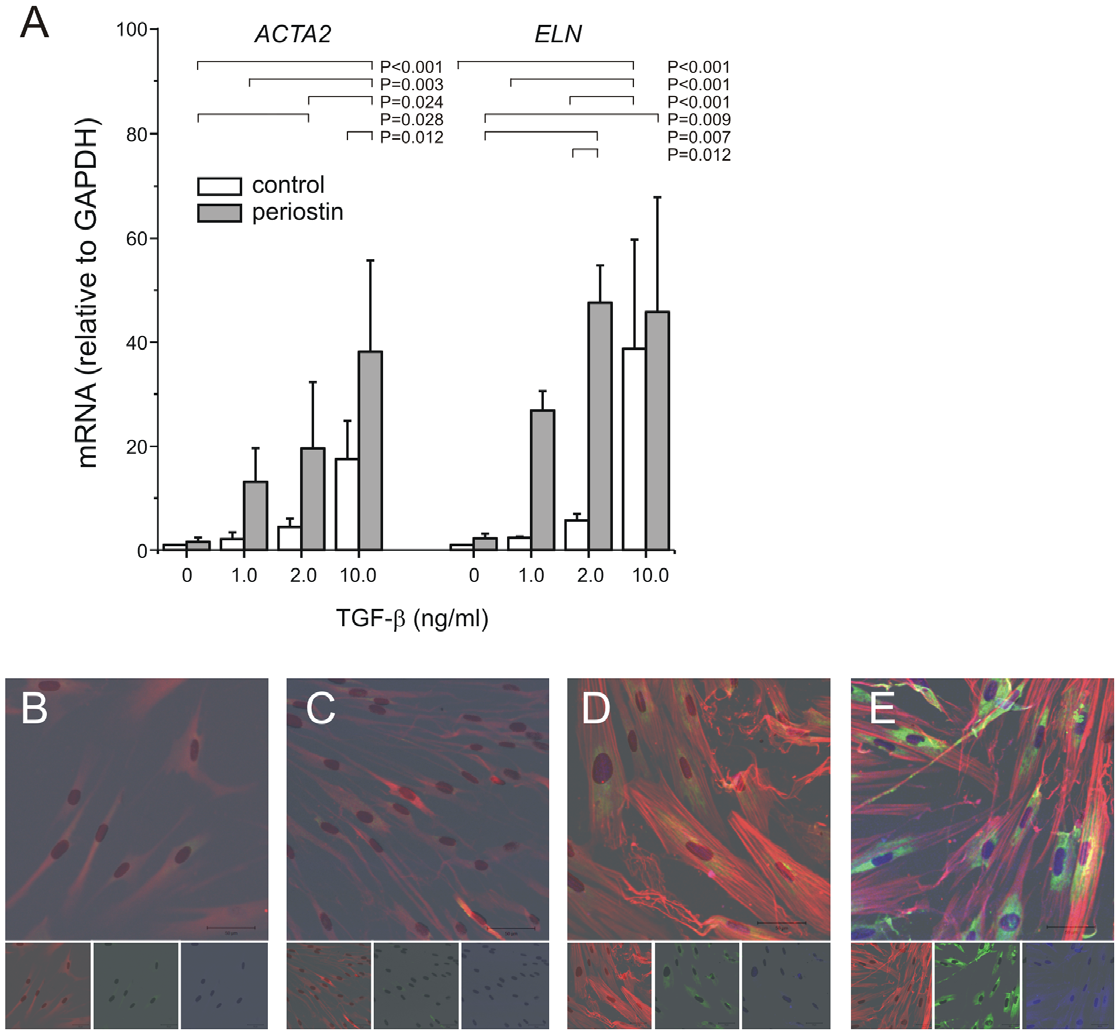
Effects of periostin and TGF-β on mesenchymal stromal cell myofibroblastic differentiation. A. Periostin (50 ng/ml) was incubated with mesenchymal stromal cells (n = 6) in the presence or absence of TGF-β (2–10 ng/ml). α-smooth muscle actin and elastin mRNA expression were measured by qPCR. We used two-way ANOVA with Fisher's least significant difference multiple comparison test to assess the individual and combined effects of TGF-β and periostin on neonatal lung mesenchymal stromal cell α-actin and elastin gene expression. TGF-β significantly increased α-actin and elastin expression in both the presence and absence of periostin. However, periostin significantly increased α-actin and elastin expression only in the presence of TGF-β. In other experiments, cells were stained with anti-α-smooth muscle actin (red), anti-collagen I (green) and anti-elastin (blue) (B, no treatment; C, periostin, 50 ng/ml; D, TGF-β, 2 ng/ml; E, periostin, 50 ng/ml and TGF-β, 2 ng/ml). Merged and individual images are shown (original magnification, 200×, results are representative of three individual experiments).

The effects of periostin (50 ng/ml) on TGF-β-induced α-smooth muscle actin and elastin mRNA expression were examined in mesenchymal stromal cells, using two-way ANOVA for statistical analysis (Figure 9A). TGF-β concentrations of 0–10 ng/ml were used. 10 ng/ml TGF-β significantly increased α-actin and elastin expression in both the presence and absence of periostin. However, 50 ng/ml periostin significantly increased α-actin and elastin expression only in the presence of TGF-β. Immunocytochemical stains showed that periostin increases TGF-β-induced α-smooth muscle actin, elastin and collagen I protein expression (Figures 9B–9E).

## Discussion

We previously characterized the gene expression profile of neonatal lung mesenchymal stromal cells [13]. Among other genes, we found that, compared to lung fibroblasts, mesenchymal stromal cells showed increased expression of *POSTN*. *POSTN* encodes periostin, a secreted non-structural extracellular matrix protein which regulates TGF-β-mediated fibrosis [19] and myofibroblast differentiation [20]. Previous studies have shown that periostin is also expressed by human bone marrow-derived mesenchymal stem cells [18], [28].

The isolation of mesenchymal stromal cells from neonatal tracheal aspirates confers a nearly 23 fold increase in the odds of developing BPD [12]. Overexpression of TGF-β in neonatal mouse lungs induces proliferation of α-actin-positive cells within the alveolar septal walls and hypoalveolarization [9], a phenotype analogous to BPD. On this basis, we hypothesized that periostin may play a prominent role in BPD pathogenesis. To test this, we measured periostin expression in mice exposed to hyperoxia during the first two weeks of life, a period which corresponds to the alveolar stage of human lung development [24]. We also examined periostin expression in the lungs of human infants with BPD. We found that periostin expression is increased in the lungs of hyperoxia-exposed neonatal mice. Periostin was present in the alveolar walls, particularly in areas of interstitial thickening associated with α-smooth muscle actin-positive myofibroblasts. In human BPD lungs, the distribution of staining was similar, with prominent staining in the subepithelium of thickened alveolar walls and fibroblastic foci. A similar distribution was recently described in patients with usual interstitial pneumonia and fibrotic-type nonspecific interstitial pneumonia [29]. The colocalization of periostin and α-smooth muscle actin suggests that periostin was expressed in part by alveolar myofibroblasts.

To further examine the possible contribution of periostin to BPD pathogenesis, we compared the responses to hyperoxia of wild-type mice to periostin null mice. As expected. wild-type mice show hypoalveolarization and interstitial fibrosis when exposed to hyperoxia during the alveolar period of lung development. Lung mRNA expression of α-actin, elastin and periostin was also increased. In contrast, periostin null mice showed normal alveolar development. Further, in contrast to control mice, the alveolar walls of periostin null mice did not show thickening with α-smooth muscle-positive myofibroblasts or deposition of collagen or elastin. Lung mRNA expression of α-actin and elastin was not increased in hyperoxia-exposed periostin null mice. These results suggest that periostin is required for hypoalveolarization and interstitial fibrosis in hyperoxia-exposed neonatal mice.

TGF-β overexpression in neonatal mouse lungs induces proliferation of α-smooth muscle actin-positive cells within the septal walls [9]. On this basis, we examined the effects of periostin on neonatal lung mesenchymal stromal cell proliferation and myofibroblastic differentiation. TGF-β and periostin had synergistic effects on DNA synthesis. Further, treatment with periostin enhanced TGF-β-induced α-smooth muscle actin, elastin and collagen type I expression, outcomes indicative of myofibroblast differentiation. These data are consistent with the notion that the observed effect of TGF-β on myofibroblast proliferation and differentiation, which contributes to interstitial thickening and fibrosis *in vivo* [9], is mediated in part by periostin expression. Since periostin null mice were protected from hyperoxia-induced hypoalveolarization, we speculate that abnormal myofibroblast proliferation and differentiation, mediated by aberrant periostin expression, is incompatible with secondary crest development.

Matricellular proteins are non-structural proteins that are secreted and sequestered in the extracellular matrix, where they interact with integrins, growth factors, proteases, cytokines and other extracellular matrix proteins. Among various functions, they regulate TGF-β activation, adhesion, migration, fiber deposition and angiogenesis, as well as cell proliferation, differentiation and survival. All are involved in fibrosis and increased matrix deposition, and most are expressed at low levels in normal adult tissue but upregulated during development, wound healing and tissue remodeling. Thus, many of the effects of TGF-β may be mediated by matricellular proteins. Few studies have examined the role of matricellular proteins in neonatal lung injury. Overexpression of connective tissue growth factor (CTGF) during development results in a lung phenotype analogous to BPD (33). CTGF expression is also increased after high tidal volume ventilation in newborn rat lungs (34). Lysyl oxidase, which crosslinks collagen and elastin, is proteolytically activated by CTGF and periostin [22], [23], and increased in the lungs of infants with BPD and hyperoxia-exposed mice (35). Since overstabilization of the extracellular matrix by excessive lysyl oxidase activity might impede the normal matrix remodeling that is required for pulmonary alveolarization, it is conceivable that the periostin knockout mice are protected from hypoalveolarization due to reduced lysyl oxidase activation.

Increasing evidence suggests that periostin plays a role in lung inflammation. Subepithelial periostin deposition and fibrosis are present in the bronchial tissue of both ovalbumin-sensitized and ovalbumin-inhaled mice and patients with asthma [21]. Periostin mRNA expression is upregulated in bronchial epithelial cells of asthmatic subjects [18], [30]. Periostin null mice show defect in allergen-induced eosinophil recruitment to the lungs [31]. In the present study, the lungs of periostin null mice showed attenuated expression of the neutrophil and monocyte chemoattractants CXCL1, CXCL2 and CCL4. CXCR2, the receptor for CXCL1 and CXCL2, is required for double-stranded RNA-induced neutrophil sequestration and hypoalveolarization in newborn mice [32], and NF-κB signaling in fetal lung macrophages disrupts airway morphogenesis [33]. Together, these data are consistent with the notion that periostin promotes hyperoxia-induced lung inflammation in neonatal mice.

There are important limitations to our study. First, the periostin null mice we used were developed in the 129 strain and backcrossed to C57BL/6. Since neither the 129 or C57BL/6 strain are perfect controls, we employed C57BL/6 mice for this purpose. Nevertheless, strain differences in the response to hyperoxia could have contributed to the protective effect of the periostin knockout [34], [35]. However, a detailed comparison of C57BL/6 and 129/Sv strains showed similar susceptibility to hyperoxia, including changes in lung neutrophils, IL-6, and expression of collagen type Iα2 and fibronectin. Further, expression of collagens type IIIα1 and IVα3 was increased after hyperoxic exposure in the 129/S strain, suggesting that these mice are capable of a fibrotic response when exposed to hyperoxia. Second, we have not completely characterized the periostin knockout mice, which appear to have a subtle pulmonary phenotype. At post-natal age 16–17, air-exposed animals showed increased alveolar macrophages and cellularity, as well as decreased expression of angiogenesis-related genes. Nevertheless, periostin null mice did not show pulmonary hypertension, as evidenced by a normal right ventricular wall thickness. This uncoupling of alveolar development and expression of angiogenesis-related genes remains unexplained, but could relate to an acceleration of alveolarization in periostin null mice.

In conclusion, we have shown that periostin expression is increased in the lungs of hyperoxia-exposed neonatal mice and infants with BPD, and is required for hyperoxia-induced hypoalveolarization and interstitial fibrosis. Our data also demonstrate that neonatal lung mesenchymal stromal cells are not only potent sources of periostin, but also respond to periostin treatment by differentiation into myofibroblasts. These data significantly extend previous results showing that an excess of TGF-β and CTGF each induce the BPD phenotype in naïve animals [8], [9], [36]. Since periostin is a downstream effector of TGF-β, periostin may represent a promising therapeutic target for BPD.

## Materials and Methods

### Ethics statement

This study was carried out in strict accordance with the recommendations in the Guide for the Care and Use of Laboratory Animals of the National Institutes of Health. The protocol was approved by the Institutional Animal Care and Use Committee of the University of Michigan Medical School (protocol ID#10397). All surgery was performed under sodium pentobarbital anesthesia, and all efforts were made to minimize suffering.

### Animal model

Two-to-three day-old wild-type C57BL/6J and B6;129-*Postn^tm1Jmol^*/J were purchased from Jackson Laboratories (Bar Harbor, ME). The periostin null mice were developed in 129 mice and backcrossed once to C57BL/6 [37]. Mice were exposed to air or 75% oxygen for 14 days using a polypropylene chamber coupled to an oxygen controller and sensor (BioSpherix, Lacona, NY) [18]. Dams were exchanged between air and hyperoxia daily. Because of periodontal disease in periostin null mice [38], wild-type and knockout animals were fed with powdered chow. After exposure, genotyping was performed by Transnetyx (Cordova, TN). Animal work was approved by the Institutional Animal Care and Use Committee.

### Mouse lung histology, fluorescence microscopy and immunohistochemistry

Lungs were perfused with 5 mM EDTA and inflated to 30 cm H_2_O pressure with 4% paraformaldehyde (Sigma-Aldrich, St. Louis, MO). Sections were stained with hematoxylin and eosin and mean alveolar chord length determined [39]. Slides were probed with fluorophore-labeled mouse anti-α-smooth muscle actin (clone 1A4, Sigma-Aldrich), rabbit anti-collagen I, mouse anti-elastin and rabbit anti-periostin (Abcam, Cambridge, MA). Other sections were probed with anti-periostin and stained with a biotinylated anti-rabbit IgG-avidin horseradish peroxidase and diaminobenzidine detection system (Vector Labs, Burlingame, CA).

### Periostin immunoblotting

Mouse lungs were washed in PBS and homogenized in a buffer containing 50 mM Tris (pH 7.5), 100 mM NaCl, 50 mM NaF, 40 mM β-glycerophosphate, 2 mM EDTA, 200 µM Na_3_VO_4_, 1% Triton X-100, 1% SDS and 1% sodium deoxycholate containing complete protease inhibitors (Roche Diagnostics, Indianapolis, IN). After centrifugation for 30 min at 4°C at 10,000×*g*, supernatant fluids were matched for protein and 30 µg/lane processed for SDS-PAGE and nitrocellulose blotting. Nitrocellulose membranes were probed with anti-periostin (Abcam, Cambridge, MA).

### Immunohistochemistry of lung tissue from infants with BPD

Human lung tissue was obtained from the University of Rochester Lung Biorepository under a protocol approved by the Institutional Review Board of Strong Memorial Hospital (Rochester, NY). Specimens were obtained from 3 infants who died in the intensive care nursery (Table 1). The diagnosis of BPD was based on premature delivery, need for chronic respiratory support, a requirement for supplemental oxygen after 36 weeks' gestation, and consistent chest radiographs, as well as pathologic tissue diagnosis at autopsy. Specimens were also obtained from infants succumbing to non-pulmonary disorders. Samples were processed within 6 hours of death.

**Table 1:** Characteristics of patients from whom mesenchymal stromal cells were isolated.

Patient	Gender	Race	Gestational Age (wks)	Birth Weight (g)	Lived	BPD
52	F	Mixed	32 3/7	1630	yes	no
56	F	Caucasian	26 6/7	1425	yes	no
58	M	Caucasian	27 6/7	1615	yes	yes
68	M	Caucasian	27	1265	yes	yes
83	F	Caucasian	24 4/7	735	yes	yes
84	M	Caucasian	27 5/7	845	yes	yes
85	F	Caucasian	28 5/7	1180	yes	yes
87	F	African-American	29 2/7	1080	yes	no
88	F	Caucasian	28 5/7	1210	yes	yes
90	M	Caucasian	28	1160	yes	yes
92	M	Caucasian	28	800	no	yes
93	M	Caucasian	28 5/7	1395	yes	no
97	M	Caucasian	31 3/7	1620	yes	no

### qPCR

Total RNA was extracted using the RNeasy Plus Mini kit (Qiagen, Valencia, CA), then transcibed to first-strand cDNA using Taqman Reverse Transcription Reagents (Applied Biosystems, Foster City, CA). First-strand cDNA was then used to quantify the expression of periostin (POSTN), α-smooth muscle actin (ACTA2), elastin (ELN), collagen type Iα1 (COLIA1), CXCL1, CXCL2, CCL4, VEGF-A, KDR/VEGFR2/Flk1, PECAM1/CD31 and the housekeeping gene glyceraldehyde dehydrogenase (GAPDH). The primers used are shown in Table 2. All primers were from IDT (Coralville, IA) except for VEGF-A, which were obtained as an assay mix from InVitrogen (Carlsbad, CA).

**Table 2:** Oligonucleotide primers used for qPCR.

*ACTA2*	5′-CCA GGC ATT GCT GAC AGG AT	5′-CCA CCG ATC CAG ACA GAG TAC
*ELN*	5′-CTG CTC CAG CTC CAA CAC CAT AGC	5′-GAG CCA GAG GTG GAG TTG GCA T
*COL1A1*	5′-AAT GGC ACG GCT GTG TGC GA	5′-AGC ACT CGC CCT CCC GTC TT
*POSTN*	5′-GCC TTA GCG ACC TCT ACA AT	5′-TAG CCG TCC GAT ACA CAA
*CXCL1*	5′-GCC ACC CGC TCG CTT CTC TG	5′-GGC ACT GAC AGC GCA GCT CA′
*CXCL2*	5′-GCC ACC CGC TCG CTT CTC TG	5′-GGC ACT GAC AGC GCA GCT CA
*CCL4*	5′-TGG CTG CCT TCT GTG CTC	5′-GCC GGG AGG TGT AAG AGA AAC
*VEGFA*	5′-GCC TCC CTC AGG GTT TCG GGA	5′-GGT GAC GAT GAT GGC GTG GTG G
	5′-CGG GCC TCG GTT CCA GAA GG	5′-CCC TCT CCG GCT CGG ACT GC
	5′-GCG CAG ACA GTG CTC CAG CC	5′-CTT GGC GGG CTC CTC TCC CT
	5′-GCG AGG CAG CTT GAG TTA AAC GA	5′-GGA GGC TCC TTC CTG CAG CC
*KDR*	5′-CAC AGG GAC CTG GCA GCA CG	5′-AAA TGT CCC GGG CCA AGC CG
*PECAM1*	5′- CGC TGC CAA GCT GGG ATC CTG	5′- AAG GAC TCC TGC ACG GTG ACG
*GAPDH*	5′-TCC ACT CAC GGC AAA TTC AAC	5′-CGC TCC TGG AAG ATG GTG ATG

### Cell culture

Isolation of neonatal lung mesenchymal stromal cells was approved by the University of Michigan Institutional Review Board. Cells were isolated from tracheal aspirates of premature infants undergoing mechanical ventilation (Table 2), as described [13]. Passage 3 and 4 cells were studied.

### Right ventricular wall thickness

Hearts from normoxic or hyperoxic mice were removed, fixed in formalin, and processed for hematoxylin and eosin staining. Images of the right ventricular outer wall were made at 10× magnification and measured using NIH ImageJ. A hemacytometer was used to standardize the number of pixels/micrometer.

### Immunocytochemistry

Mesenchymal stromal cells were grown on fibronectin-coated glass slides (BD Biosciences), fixed in 1% paraformaldehyde and probed with antibodies against α-smooth muscle actin, collagen I and elastin.

### [^3^H]-thymidine incorporation

Mouse lung mesenchymal cells were plated into 12-well dishes at 100,000 cells/well, allowed to attach overnight, and incubated in serum-free medium supplemented with 500,000 cpm [^3^H]-thymidine. After 8 h, cells were treated with TGF-β1 or mouse periostin. Cells were maintained in the medium for 48 h. At the end of this time, the radioactive medium was removed, wells were washed three times with PBS, and the DNA precipitated with 10% trichloroacetic acid at 4°C. After 30 min, the acid was removed and the precipitate was washed three times with 70% ethanol at 4°C and solubilized at room temperature for 30 min with 1 mL 0.5 N NaOH and 1% Triton X-100. Solubilized DNA was counted in a liquid scintillation counter.

### Periostin ELISA

Cell supernatants were analyzed for soluble periostin by ELISA (Adipo Bioscience, Santa Clara, CA).

### Statistical analysis

All data were described as mean±SEM. For simple comparisons of two or more groups, we used one-way analysis of variance (ANOVA). When examining the influence of two factors, we used two-way ANOVA. Significant differences between groups were pinpointed by Fisher's least significant difference multiple comparisons test.
