# Correction: Neonatal Periostin Knockout Mice Are Protected from Hyperoxia-Induced Alveolar Simplication

**DOI:** 10.1371/journal.pone.0130369

**Published:** 2015-06-11

**Authors:** Paul D. Bozyk, J. Kelley Bentley, Antonia P. Popova, Anuli C. Anyanwu, Marisa D. Linn, Adam M. Goldsmith, Gloria S. Pryhuber, Bethany B. Moore, Marc B. Hershenson

The word “Simplification” is misspelled in the title of the article. The correct title is: Neonatal Periostin Knockout Mice Are Protected from Hyperoxia-Induced Alveolar Simplification. The correct citation is: Bozyk PD, Bentley JK, Popova AP, Anyanwu AC, Linn MD, Goldsmith AM, et al. (2012) Neonatal Periostin Knockout Mice Are Protected from Hyperoxia-Induced Alveolar Simplification. PLoS ONE 7(2): e31336. doi:10.1371/journal.pone.0031336
